# Widespread Circulation of Tick-Borne Viruses in Virginia—Evidence of Exposure to Heartland, Bourbon, and Powassan Viruses in Wildlife and Livestock

**DOI:** 10.3390/microorganisms12050899

**Published:** 2024-04-30

**Authors:** Ahmed Garba, Jennifer Riley, Kevin K. Lahmers, Gillian Eastwood

**Affiliations:** 1 Department of Entomology, College of Agriculture and Life Sciences, Virginia Tech, Blacksburg, VA 24061, USA; ahmedg@vt.edu; 2 Blue Ridge Wildlife Center, Boyce, VA 22620, USA; drjen@blueridgewildlifectr.org; 3 Department of Biomedical Sciences and Pathobiology, Virginia-Maryland College of Veterinary Medicine, Blacksburg, VA 24061, USA; klahmers@vt.edu; 4 Center for Emerging Zoonotic and Arthropod-Borne Pathogens (CeZAP), Virginia Tech, Blacksburg, VA 24061, USA; 5 The Global Change Center, Virginia Tech, Blacksburg, VA 24061, USA

**Keywords:** Powassan virus, Bourbon virus, Heartland virus, *Ixodes scapularis*, *Amblyomma americanum*, wildlife, livestock, serology, tick, tick-borne diseases

## Abstract

Emerging tick-borne viruses such as Powassan virus (POWV), Bourbon virus (BRBV), and Heartland virus (HRTV), whilst rare, can cause severe health problems in humans. While limited clinical cases have been reported thus far in Virginia, the presence of tick-borne viruses poses a serious health threat, and the extent of their prevalence in Virginia is unknown. Here, we sought evidence of POWV, BRBV, and HRTV exposure in Virginia via a serological assessment of wildlife and livestock. Wildlife in Virginia were found to be seropositive against POWV (18%), BRBV (8%), and HRTV (5%), with western and northern regions of the state having a higher prevalence. Multiple wildlife species were shown to have been exposed to each virus examined. To a lesser extent, cattle also showed exposure to tick-borne viruses, with seroprevalences of 1%, 1.2%, and 8% detected in cattle against POWV, BRBV, and HRTV, respectively. Cross-reactivity against other known circulating mosquito-borne flaviviruses was ruled out. In conclusion, there is widespread exposure to tick-borne viruses in western and northern Virginia, with exposure to a diverse range of animal populations. Our study provides the first confirmation that HRTV is circulating in the Commonwealth. These findings strengthen the existing evidence of emerging tick-borne viruses in Virginia and highlight the need for public health vigilance to avoid tick bites.

## 1. Introduction

Ticks (families Argasidae and Ixodidae) are blood-feeding ectoparasites responsible for many vector-borne disease cases worldwide [1]; they can transmit a variety of pathogens to vertebrate hosts, including bacteria, protozoa, and viruses [2,3]. Tick-borne pathogens are currently responsible for about 95% of all vector-borne disease incidences in the United States (US) [3]. Tick-borne diseases threaten human and animal lives and have a significant economic impact due to the costs associated with their control, treatment, or post-treatment disabilities [1]. Lyme disease, caused by the bacterium *Borrelia burgdorferi*, is the most prevalent and well-known tick-borne illness in the US [1,4], and the cost of overall patient care for Lyme disease is estimated to be USD 1 billion annually [5]. Babesiosis, ehrlichiosis, anaplasmosis, and Rocky Mountain spotted fever are further tick-borne illnesses expanding in distribution and prevalence in the US, and importantly, infections caused by tick-borne viruses represent an emerging threat to public health. Despite efforts to reduce the burden of tick-borne diseases, there has been a significant rise in the occurrence of these diseases over the past two decades [6]. Factors promoting their incidence include expanding tick ranges, climate change, a lack of vaccines for endemic tick-borne diseases, and the emergence of novel tick-borne pathogens [7]. In addition, invasive or new tick species are being detected in novel territories with expanding populations [2].

Emerging tick-borne viruses are rare etiological agents but can pose a more severe threat to human health than non-viral agents, with no specific therapy or vaccine available, and consequences of infection including death or ongoing neurological abnormalities in survivors [8,9,10]. In the US, three emergent tick-borne viruses in particular, Powassan virus (POWV), Bourbon virus (BRBV), and Heartland virus (HRTV), have been isolated from ticks, as well as an occurrence of human cases in several states, highlighting the potential threat of these viruses to public health. In Virginia, there has been one human case each of POWV (Centers for Disease Control and Prevention [CDC], 2023) and likely HRTV [11]. However, there is currently a lack of a wider understanding of POWV, BRBV, or HRTV in Virginia.

Powassan virus is a rare but fatal neurotropic, tick-borne flavivirus (family *Flaviviridae*), having an enveloped positive-sense, single-stranded RNA genome [8]. First isolated from the brain of a fatal pediatric case in Ontario, Canada, in 1958. POWV is the only member of the tick-borne encephalitis serogroup in North America [12]. POWV infections are characterized by fever, headache, ataxia, encephalitis, and meningitis [13]; the symptoms of POWV in non-human vertebrates are currently unknown. There are two serologically indistinguishable lineages of POWV. Lineage I (POWV-I) is primarily associated with the groundhog tick (*Ixodes cookei*) and the squirrel tick (*I. marxi*) [14]. Lineage II (POWV-II) or deer tick virus (DTV) is associated with the blacklegged tick (*I. scapularis*) [15]. Since *I. scapularis* is a human-biting tick and *I. marxi* or *I. cookei* infrequently feed on humans, human exposure to POWV is generally associated with *I. scapularis*, whose distribution extends predominantly into the eastern states of the US, including Virginia [8,16]. Most human cases of POWV have historically been reported in the Midwest or Northeastern regions of the country, despite the tick vectors’ distribution extending over a further geographical range. In Virginia, *I. scapularis* is present [17], particularly in Appalachian counties, yet little is known about the prevalence of POWV. The molecular detection of POWV RNA in *I. scapularis* ticks collected in Southwest Virginia was recently documented [18], and a confirmed human case of POWV was thought to have been contracted in Virginia (Franklin County) in 2009 (CDC, 2023).

A second emerging tick-borne virus in the US is HRTV. HRTV is a novel tick-borne bandavirus (family *Phenuiviridae*), having a tri-segmented, single-stranded negative-sense RNA genome. This virus is genetically closely related to severe fever with thrombocytopenia syndrome virus (SFTSV) that causes mortality and morbidity in Asia [19,20]. First isolated and identified in two separate human cases involving Missouri farmers in 2009 [20], HRTV infections are characterized by fever, leukopenia, and thrombocytopenia; symptoms of HRTV in non-human vertebrates are currently unknown. HRTV is vectored by *Amblyomma americanum* (the lone star tick) [21]. Since the first index case in 2009, over 60 new clinical cases of HRTV have been reported, mostly in the US states of the South and Midwest according to the CDC. One fatal human Heartland infection case was tentatively attributed to a tick bite in Virginia (possibly Maryland) [11], and viral RNA of HRTV has been detected in *A. americanum* collected in the Commonwealth (Our lab has detected personnel communication/in prep; Eastwood, 2024 [22]); otherwise, little is known about the circulation of HRTV in the state.

A third emerging tick-borne virus in the US is Bourbon virus (BRBV). This thogotovirus (family *Orthomyxoviridae*; single-stranded negative-sense RNA) was first detected in a blood sample collected in a fatal case involving an adult male (over 50 years) resident of Bourbon County, Kansas, US, in 2014 [23]. BRBV is the first thogotovirus to be identified in the US with the ability to cause disease and death in humans, and infection is characterized by fever, leukopenia, and thrombocytopenia [9,24,25]; symptoms of BRBV in non-human vertebrates are currently unknown. BRBV uses *A. americanum* as its arthropod vector [23]. Despite sharing the same tick vector with HRTV, which has a wide distribution, fewer human cases of BRBV (only five, with two fatalities) have been reported so far in the US. Cumbie et al. [26] identified the presence of BRBV viral RNA in *A. americanum* and *Haemaphysalis longicornis* ticks collected in several western counties of Virginia. That study also detected neutralizing antibodies (NAbs) against BRBV in two wildlife species (white-tailed deer and norther raccoon) from the same region. Despite this, there remains a lack of understanding regarding the distribution of the virus in a broader region of Virginia and BRBV-exposure in other species.

In general, most human cases of POWV, BRBV, and HRTV have been restricted to the geographical range of their primary tick vectors (*I. scapularis* or *A. americanum*) where these ticks are abundant. However, populations of these two tick species are expanding both in abundance and geographically in general terms to north and south, respectively [2,27]. Thus, additional regions of the US have the potential for tick-borne viruses to emerge in them. Vertebrates in these virus-endemic regions with a tick presence could be exposed [27,28,29] and may contribute to the transmission of POWV, BRBV, and HRTV as a reservoir or amplification host or may gain infection as a dead-end host. Tick-borne viruses are believed to be maintained in an enzootic cycle between diverse small-to-medium-sized vertebrate host species and competent tick vectors [30], albeit that non-viremic localized (tick co-feeding) transmission may also occur, on a host without the host acting as a reservoir. To effectively monitor the spread of POWV, BRBV, and HRTV and ascertain the current geographical distribution and prevalence in the US, serology can be used to identify circulation and exposure in vertebrate species. The serological surveillance of NAbs has been used in virus-endemic or emergent areas to assess pathogen distribution and exposure to resident wild and domestic animals; such methodology can also be used to monitor arboviruses within a known arboviral ecology [31,32,33]. Indeed, NAbs against POWV, BRBV, and HRTV have been detected in both wild (deer, raccoons, groundhogs, red squirrels) and domestic animals (dogs, horses) in different parts of the US—notably, in areas where human cases of these three tick-borne viruses were reported [29,34,35,36,37]. This current study describes the first comprehensive serosurveillance of livestock and wildlife species in Virginia, and we reveal widespread exposure to POWV, BRBV, and HRTV.

## 2. Materials and Methods

### 2.1. Sample Collection

**Figure 1:**
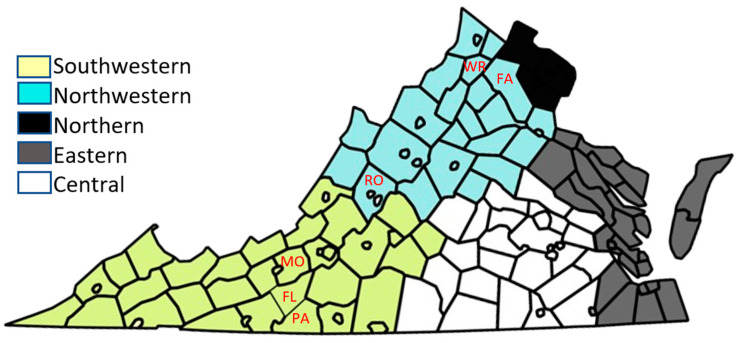
Map of Virginia showing the five health planning regions. The six counties marked (Montgomery (MO), Rockbridge (RO), Fauquier (FA), Warren (WR), Patrick (PA), and Floyd (FL) counties) were areas of active surveillance for wildlife sampling.

Both wildlife and livestock from across Virginia’s five health planning regions (HPRs; Figure 1) were assessed during the study. For wildlife, both passive and active surveillance techniques were utilized to collect blood samples from wildlife between July 2020 and November 2022. First, in passive surveillance, targeting mammals but including a broad range of wild vertebrate species, blood samples were collected from (i) the body cavity of deer brought to Virginia Department of Wildlife Resources (DWR) check stations for chronic wasting disease surveillance in Virginia (Fall 2021 and 2022), (ii) a variety of wild species presented to rehabilitation centers in northern and western Virginia, and (iii) a variety of wild mammalian species as recently killed animal carcasses (roadkill samples) and hunter donations. Second, active surveillance involved small mammal trapping targeting white-footed mice (*Peromyscus leucopus*), using seed-baited modified Fitch traps and Sherman live traps, in Montgomery (MO), Rockbridge (RO), Fauquier (FA), Warren (WR), Patrick (PA), and Floyd (FL) counties (indicated within Figure 1). About 40–50 traps were set for an average of three nights per month, resulting in a total trapping effort of 456 h. A blood sample was taken from the captured target species; briefly, a sterile 5.0 mm lancet was used to obtain 5–40 µL of blood (depending on the size of the animal) via submandibular venipuncture, transferred into capillary tubes (Virginia DWR Scientific Collection Permit #069872, IACUC#20-197). After the sample collection, each captive was marked using ear tags, observed for 10 min for signs of stress or shock, and then released in the same capture location. Blood samples, from all species tested, were transported to the laboratory at Virginia Tech in a cool box, and serum was separated via centrifugation at 5000 rpm for 6 min and then transferred to a fresh sterile tube.

**Figure 2:**
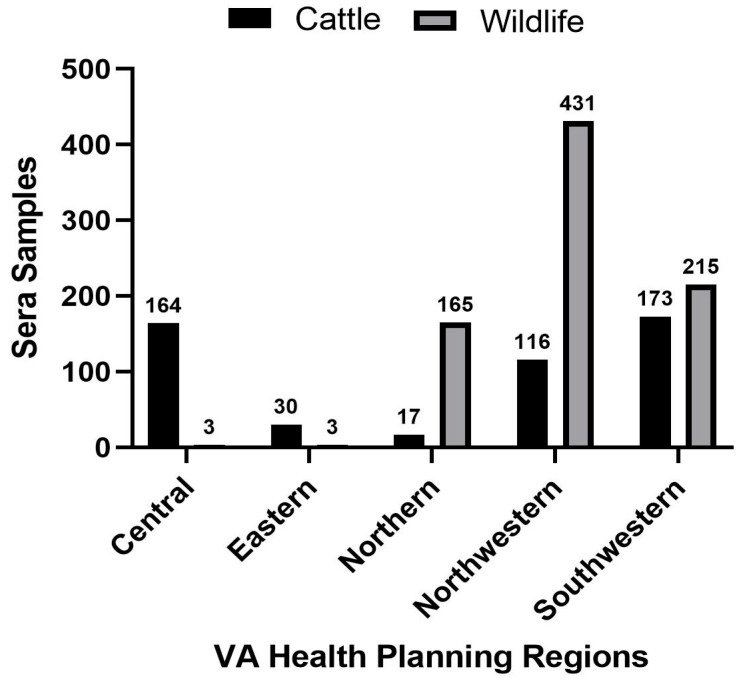
Cattle and Wildlife sera samples available across each of Virginia’s Health Planning Regions.

For livestock sampling, a total of 500 cattle serum samples were provided from an archive collection held at the Department of Biomedical Science and Pathology in the Virginia-Maryland College of Veterinary Medicine at Virginia Tech (IACUC#18-143). The cattle samples originated from various breeds and animal ages, from both local auctions and private owners, across all HPRs in Virginia (indicated in Figure 2) between 2019 and 2022.

### 2.2. Serological Assessment

Prior to assessment, aliquots of sera samples were heated at 56 °C for 30–60 min to inactivate cell growth inhibitors in culture and then diluted 1:20 in Dulbecco’s Modified Eagle Medium (DMEM) (Gibco, Billings, MT, USA) supplemented with 2% fetal bovine serum (FBS) (Gibco), 1% penicillin-streptomycin (Gibco), and 0.037% sodium bicarbonate. Plaque reduction neutralization tests (PRNT), as described by Eastwood et al. [38], were used to screen all sera samples for the presence of NAbs against each tick-borne virus of interest. Briefly, the diluted sera were challenged against a virus suspension at a working concentration of 800 PFU/mL of either HRTV (MO-4 strain, BEI Resources; propagated and stock titered at 3 × 10^7^ PFU/mL), BRBV (Original strain, BEI Resources; 2 × 10^8^ PFU/mL), or POWV (DTV-West Nile virus (WNV) chimeric virus (DTV-prME/WNV), kindly provided by Greg Ebel, Colorado State University; 3.1 × 10^7^ PFU/mL). Seropositive samples demonstrating at least an 80% reduction in plaque formation were confirmed by serial twofold dilution to establish the minimum antibody neutralization titer (end-point titer). Only seropositive samples with antibody titers ≥ 40 were considered seropositive. Rabbit antisera (1:10 dilution) (kindly provided by the US Centers for Disease Control and Prevention, Atlanta, GA, USA) was used as a positive serum control, while DMEM and a no-template control were used as negative controls for the assays.

### 2.3. Statistical Analysis

Seroprevalence rates or the proportion and their corresponding confidence intervals (Cis) were calculated using the R studio software (R version 4.3.2). The observed proportions were compared, and the statistical significance of the test results was evaluated through Chi-Square Tests (χ^2^), employing a null hypothesis that there is no significant difference in the wildlife and cattle seroprevalence. This analysis employed a 95% confidence interval for all positive test outcomes.

## 3. Results

### 3.1. Overall Seroprevalence

A total of 836 wildlife serum samples from different age classes (432 adults, 113 juveniles, 37 infants, 254 unreported) were collected for this study across all five HPRs in Virginia (locations indicated in Figure 2; *n* = 19 samples were from an unknown HPR and further excluded from the HPR analysis); Supplementary Table S1 lists all species. Due to the sample volume, not every individual could be tested for its sero-status against all the tick-borne viruses of interest. Of the 811 individuals tested for POWV Nab, 144 (18%) were seropositive, while 61 (8%) of the 770 individuals tested against BRBV were seropositive, and 38 (5%) out of the 766 individuals tested for HRTV were seropositive (Table 1; Supplementary Table S2 shows a breakdown of wildlife serostatus by health region).

A total of 500 cattle sera were screened for NAbs against POWV, BRBV, and HRTV, of which 5 (1%) were found to have neutralizing antibodies against POWV, 6 (1.2%) samples showed NAbs against BRBV, and 40 (8%) samples had NAbs against HRTV (Table 2; Supplementary Table S3 shows a breakdown of cattle serostatus by health region). The serotiters of seropositive samples of POWV, BRBV, and HRTV ranged from 1:40 to 1:≥320 for each virus. Our analysis revealed substantial differences in the seroprevalence rates against Powassan virus (χ^2^ = 84.55, *p* < 0.05), Bourbon virus (χ^2^ = 26.08, *p* < 0.05), and Heartland virus (χ^2^ = 4.32, *p* < 0.05), contrasting wildlife and cattle livestock.

### 3.2. Seroprevalence by Wildlife Species

**Figure 3:**
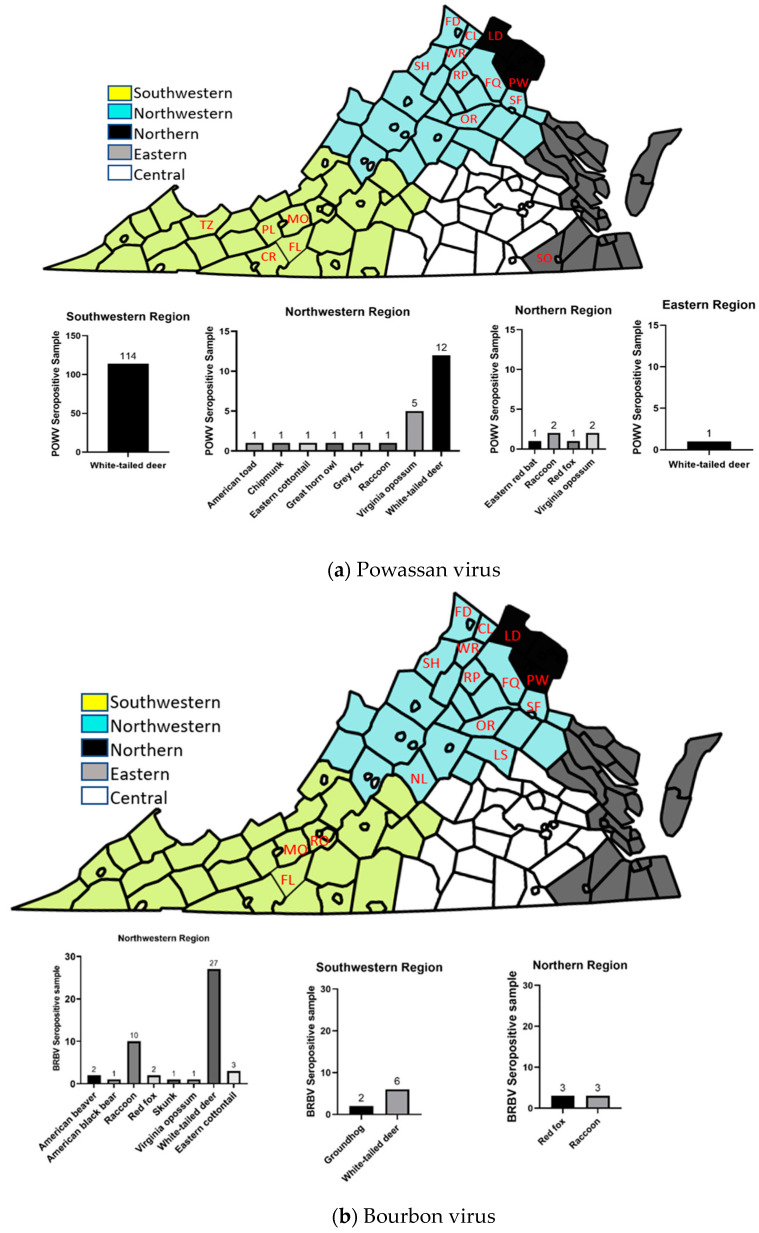

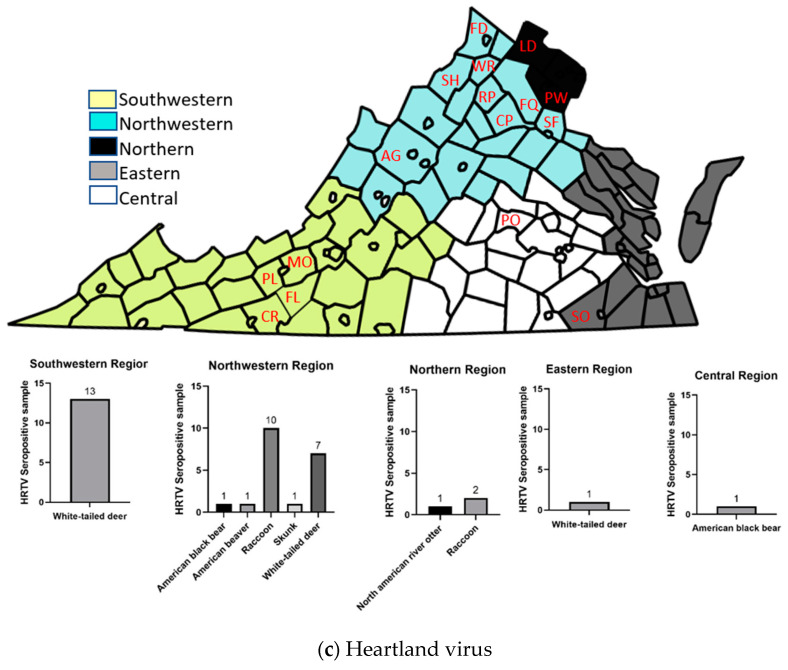
Maps of Virginia and species bar charts showing the composition and distribution of virus-seropositive wildlife samples across Virginia’s Health Planning Regions, with Counties marked to indicate the source of seropositive samples (a) Powassan virus; (Carroll (CR), Clarke (CL), Fauquier (FQ), Floyd (FL), Frederick (FD), Loudoun (LD), Montgomery (MO), Orange (OR), Prince William (PW), Pulaski (PL), Rappahannock (RP), Shenandoah (SH), Southampton (SO), Stafford (SF), Tazewell (TZ), Warren (WR)). (**b**) Bourbon virus; (Clarke (CR), Fauquier (FQ), Floyd (FL), Frederick (FD), Loudoun (LD), Louisa (LS), Montgomery (MO), Nelson (NL), Orange (OR), Prince William (PW), Rappahannock (RP), Roanoke (RO), Shenandoah (SH), Stafford (SF), Warren (WR)) (**c**) Heartland virus; (Augusta (AG), Carroll (CR), Culpeper (CP), Fauquier (FQ), Floyd (FL), Frederick (FD), Loudoun (LD), Montgomery (MO), Powhatan (PO), Prince William (PW), Pulaski (PL), Rappahannock (RP), Shenandoah (SH), Southampton (SO), Stafford (SF), Warren (WR)).

Table 3 details the presence of neutralizing antibodies (NAbs) in various wildlife species (N > 5 individuals tested) for each virus. Specifically, against POWV, NAbs were observed in five different wildlife species (N > 5). In addition, a single individual available from each of five further species (*Anaxyrus americanus*, *Tamias striatus, Urocyon cinereoargenteus, Lasiurs borealis*) tested POWV-seropositive. For BRBV, NAbs were detected in six different wildlife species (N > 5), with three further species (two of three *Marmota monax* tested, both *Castor canadensis* tested, and one of four *Ursus americanus*) showing seropositivity against this virus. Finally, for HRTV, NAbs were found in four distinct wildlife species (N > 5), with an additional two species (the single *Lontra canadensis*, and one of two *Castor canadensis*) also HRTV-seropositive against HRTV. The seropositive wild animals were distributed throughout the five Virginia health regions, as depicted in Figure 3a (POWV), 3b (BRBV), and 3c (HRTV).

### 3.3. Regional Seroprevalence

#### 3.3.1. Wild Animals

Considering the POWV seroprevalence in the different HPRs of Virginia, in the Northwest, 23 out of 429 individuals tested were found to be seropositive against POWV, representing a prevalence of 5.4% (95% CI: 3.2–7.5%). In the Northern HPR, 6 out of 164 wildlife individuals tested showed seropositivity at a rate of 3.7% (95% CI: 0.8–6.5%). In the Southwestern HPR, 114 were POWV seropositive out of 212 individuals tested (54%; 95% CI: 47–60%). Limited sample sizes were available from both the Central and Eastern HPR, three individuals each, and there were no seropositive cases in Central HPR, while in the Eastern region, one out of three samples was seropositive (33%; 95% CI: 0–87%).

Considering BRBV seroprevalence, the data revealed similar regional disparities. In the Northwestern HPR, 47 out of 423 individuals tested were BRBV-seropositive, indicating an 11% (95% CI:8.1–14.1%) prevalence rate in local wildlife. In the Northern region, 6 out of 165 individuals (3%; 95% CI: 0.4–5.6%) tested seropositive against BRBV. The Southwestern HPR showed 8 seropositive wildlife out of 176 individuals tested (4.5%;95% CI: 1.5–7.6%). Neither of the Central and Eastern regions exhibited BRBV-seropositivity among the limited sample size of three individuals each.

Considering the HRTV seroprevalence across the HPRs, from the Northwestern region, 20 out of 422 individuals tested were found to be seropositive for HRTV (4.7%; 95% CI; 2.7–6.8%). From the Northern region, 3 out of 164 individuals were HRTV-seropositive (1.8%; 95% CI; 0–3.9%). A total of 13 seropositive cases out of 174 individuals tested were observed from the Southwestern HPR (7.5%; 95% CI: 3.6–11.4%). Of the three wildlife samples available from both the Central and Eastern regions, one individual from each showed seropositivity against HRTV (33%, 95% CI: 0–87%).

#### 3.3.2. Livestock (Cattle)

Cattle samples originated in multiple HPRs, with 116 samples from the Northwestern region, 17 from the Northern region, 173 from the Southwestern region, 164 from the Central region, and 30 from the Eastern region.

Considering POWV, out of the five seropositive cattle detected, one sample was from the Northwestern HPR, indicating a seropositivity rate of 0.9% (95% CI: 0–2.5%). The Southwestern HPR demonstrated four seropositive cattle samples with a seroprevalence of 2.3% (95% CI: 0.1–4.6%). Meanwhile, no seropositivity against POWV was detected in samples from the Northern, Central, and Eastern HPRs.

Considering BRBV exposure, detected in 6 cows in total, the following variations were recorded. In the Northwestern region, three cattle samples were BRBV-seropositive, a prevalence rate of 2.6% (95% CI: 0–5.5%). No NAbs to BRBV were detected in the samples tested from the Northern and Southwestern HPRs. In the Central region, two seropositive samples were detected (1.2%; 95% CI: 0–2.9%), while one seropositive sample, or 3.3% (95% CI: 0–9.9%) seroprevalence, was detected in the Eastern HPR.

Heartland virus exposure in cattle was detected in all the HPRs, as evidenced by HRTV seropositivity in 40 individuals. The highest seroprevalence rate was detected in the Central region followed by the Northwestern region, Southwestern region, Eastern region, and Northern region, respectively. In the Central region, 24 HRTV-seropositive samples, representing a prevalence of 15% (95% CI: 9.2–20), were recorded. Samples from the Northwestern region yielded nine seropositive samples (7.6% (95% CI: 2.9–13%)). Four seropositive samples came from the Southwestern region (2.3%; 95% CI: 0.1–4.6%), while the Eastern and Northern regions provided two and one seropositive samples, with seroprevalence rates of 6.7% (95% CI: 0–16%) and 5.9% (95% CI: 0–17%), respectively.

## 4. Discussion

Our research aims to assist in understanding the emergence and current status of three tick-borne viruses in Virginia. Novel pathogens in a new region pose a potential threat to public or animal health, and understanding the extent of their geographical spread is crucial to reducing disease burden. This information is critical for public health officials and researchers to understand arbovirus emergence and determine the best control strategies for tick-borne viruses. Here, we examined immune responses developed for each of the three arboviruses among wild and livestock animal populations. By understanding the rate of exposure in vertebrates, we can gauge the activity of transmission and better understand the ecology of tick-borne viruses and where they circulate in novel emergent regions such as Virginia. We determined the prevalence of three tick-borne viruses circulating in the Commonwealth. POWV, BRBV, and HRTV are rare but serious arboviruses emerging in the US, with limited knowledge as to how established they had become in Virginia and the extent to which they might be circulating. All three tick-borne viruses under focus have the potential to cause serious clinical disease or even mortality in humans, with further research needed as to their effect on wildlife or livestock. Our results provide evidence that all three viral pathogens are circulating in Virginia, with past exposure to POWV, BRBV, and HRTV observed in species of wild animals as well as in domestic cattle from multiple HPRs in Virginia. These investigations reveal the first detection of NAbs against HRTV and POWV in vertebrates in this region and suggest the need to better understand the ecological dynamics of emerging tick-borne viruses. These findings have important implications for public health and highlight that tick-borne virus surveillance for identifying disease risk areas is warranted.

This study provides the first evidence of HRTV circulation in the Commonwealth. We crucially detected neutralizing antibodies against HRTV in all five HPRs of Virginia. Neutralizing antibodies against POWV were detected in all HPRs except for the Central region of Virginia, similarly indicating widespread viral activity in this state. Our findings further identified new areas of BRBV circulation in Virginia by reporting exposure to the virus in Northwestern and Northern regions. Earlier research had reported NAbs against BRBV in white-tailed deer, raccoon, and groundhogs in the Southwestern region of Virginia [26]. Our current study corroborates that finding by detecting NAbs against BRBV in white-tailed deer, as well as evidencing the exposure of additional wildlife species—red fox, American black bear, American beaver, skunk, eastern cottontail, and Virginia opossum—and showing that circulation occurs in the Northern and Northwestern regions of Virginia. We also reveal serological evidence of POWV and HRTV in white-tailed deer in Southwest Virginia. These findings support the hypothesis that these emerging tick-borne viruses are circulating in Virginia. Detecting neutralizing antibodies in multiple animal species and age classes across all Virginia HPRs suggests a widespread exposure and possible geographical expansion of POWV, BRBV, and HRTV in Virginia.

### 4.1. Species Exposure to Each Tick-Borne Virus

The present study recorded species variation in exposure to the three tick-borne viruses: First, for POWV, our research found a seroprevalence of 49.8% (*N* = 255) among white-tailed deer, which is higher than the seroprevalence of any other vertebrate species, in which *N* ≥ 98 is needed to detect true POWV seroprevalence (95% confidence) in wildlife species based on our sample size calculations (power analysis) using reported seroprevalence in previous studies. The high exposure in white-tailed deer may be due to a strong association between this host and adult-stage *I. scapularis* ticks. This finding further reinforces the notion that white-tailed deer (*O. virginianus*) are often exposed to POWV, as reported by Nofchiessy et al. for New England [39], although this does not imply that the species necessarily play a role in POWV transmission dynamics. Our research also reports POWV exposure in vertebrate species such as the Eastern cottontail and American black bear, thus expanding the list of potential species encountering this virus. Although the main focus of the study was on mammals, a small subset of avian species were available to test, and an interesting discovery was the detection of NAbs against POWV in one bird species tested, the great horned owl, a resident species of Virginia, suggesting local exposure. This discovery is in line with Dupuis et al. (2013) [28], who detected NAbs against POWV in each of the Northern Cardinal (*Cardinalis cardinalis*), Gray Catbird (*Dumetella carolinensis*), and Eastern Towhee (*Pipilo erythrophthalamus*) and Veery (*Catharus fuscescens*) while sampling avian hosts in New York state, albeit at low rates (4/727 total screened). The specific role of birds in POWV transmission is not clear, and the presence of neutralizing antibodies from an immune response does not imply that the virus can replicate or transmit from a particular host. Although *I. scapularis* is known to feed on birds, exposure to POWV may not always be through the primary tick vector. Therefore, it is critical to intensify tick surveillance efforts to identify non-primary tick vectors that may contribute to the transmission and geographical expansion of the pathogen in Virginia. Further research is necessary to fully decipher any role of the species discussed.

Considering HRTV, previous studies by Riemersma and Komar [31] and Bosco-Lauth et al. [29] reported raccoons and white-tailed deer as species that might be vital for HRTV serosurveillance. Our study supports this hypothesis regarding the exposure of these species to the virus, although it found a higher seroprevalence in raccoons (10.7%) than in white-tailed deer (8.5%). These Virginia rates of exposure are less than in Missouri (42% and 14%, respectively, for these species), likely related to Missouri being the location of the index case of the Heartland virus emergence [29]. Here, we show that other wildlife species present in Virginia, such as the American black bear, skunk, American beaver, and one North American river otter, have also been exposed to this bandavirus. These findings expand the list of vertebrates known to have been exposed to HRTV and which mount an immune response. However, additional research is needed to better understand the virus ecology and its potential impact on exposed animals and define any host role.

Third, for BRBV, a wide range of wildlife species were similarly seropositive. As mentioned above, these findings corroborate earlier reports of white-tailed deer, groundhogs, and raccoon in Virginia being exposed to BRBV [26]. In support of serosurveys conducted in Missouri and North Carolina [34,37], white-tailed deer and raccoons remain the most exposed wildlife species to BRBV, with comparable rates (56% seroprevalence in white-tailed deer) in neighboring North Carolina [37]. This species may be useful as wildlife sentinel candidates in tracking pathogen spread, since all life-stages of *A. americanum*, the key tick vector of BRBV, feed on deer, implying a high risk of exposure.

### 4.2. Recent Viral Activity

In terms of the wildlife population structure, all age-classes were exposed to POWV, BRBV, and HRTV, with a majority of known seropositive samples being adults. However, the detection of NAbs in 20 known juveniles and infants - from the Northwestern (POWV = 2, BRBV = 11, HRTV = 4), Northern (HRTV = 2), and Central (HRTV = 1) Virginia health planning regions, if not maternally induced, strongly suggests ongoing viral circulation and/or activity within Virginia, as antibodies in young individuals typically indicate recent exposure. The prevalence of these viruses might be pronounced in certain areas. Indication of recent activity in the Northwestern region may warrant further investigation in order to better understand the ecological factors contributing to the heightened viral activity.

### 4.3. Methodology and Interpretation

There are both strengths and limitations to serological test methodologies. The detection of NAbs only indicates previous viral exposure and not a current viral infection, replication, or presence in the host; the serology also cannot reveal the timing of when the infection occurred, although we can look to the age class of an individual to demonstrate possible recent transmission occurring, when young animals are seropositive, in the absence of maternally derived antibodies. Due to reports of no cross-reactivity in some wildlife species between HRTV and BRBV and their closely related viruses [29,31,40], no comparative test was performed. Specifically, BRBV is associated with the Lone star virus and Sunday Canyon virus, while HRTV is linked to the Dhori virus, Thogoto virus, and Aransas virus. However, it is possible that some cross-reactive viruses could exist, and furthermore, cross-reactivity has not been determined in all vertebrates, including cattle. In contrast, high cross-reactivity has been reported among flaviviruses. We thus subsequently tested all POWV-seropositive samples against other flaviviruses possibly circulating in the study region, namely, West Nile virus (WNV) and St. Louis encephalitis virus (SLEV), both mosquito-borne viruses. All POWV-seropositive samples were confirmed with a fourfold greater titer in these follow-up cross-neutralization tests with WNV and SLEV; two wildlife samples (one Virginia opossum and one American black bear) demonstrated notable cross-reactivity with WNV, with titers of 1:320 against WNV compared to their initial POWV titers of 1:40 and 1:80. Consequently, these two samples were concluded to not have been exposed to POWV and were thus excluded from the results of seropositivity against tick-borne viruses presented in this paper.

Sero-negative samples cannot necessarily be interpreted as naïve, as there is no clear knowledge of the duration of the antigen-antibody response and limited consistent data on the ability of an animal to seroconvert. To address the host role and the impact that tick-borne viral infection has on non-human vertebrates, susceptible host competency tests are needed to assess the viremic potential of key animal species. This would help in identifying potential vertebrate species with viremic possibilities that may serve as amplification or reservoir hosts or others that could serve as sentinels or indicator species for monitoring the spread of emerging tick-borne viruses. Bosco-Lauth et al. [41] conducted a host susceptibility test, which showed that not all exposed vertebrates, such as chickens and rabbits, developed detectable antibody responses. This finding raises important questions about detecting NAbs in exposed vertebrates. It suggests that some vertebrates without neutralizing antibodies may still have been exposed to the virus or could result in a lower seroprevalence. Despite its inability to identify potentially exposed but non-seropositive hosts, the plaque reduction neutralization test (PRNT) nevertheless remains the gold standard for arbovirus surveillance.

### 4.4. Heterogeneity in Exposure

This study represents the first examination of livestock exposure to these three novel tick-borne viruses in Virginia. Although the pathogenicity of these viruses has not been established in livestock, and seroprevalence rates were low, they are nevertheless being exposed to tick-borne viruses or cross-reactive viruses, as revealed here. Although no associated diseases have ever been reported in cattle, it would be informative to investigate the potential impact of these viruses on livestock health and reproduction, as well as their capacity to play a role in the transmission of these viruses. Surprisingly, in contrast to exposure in wildlife species, there was a higher HRTV seroprevalence compared to the other two tick-borne viruses in livestock, with the majority (24 out of 40 HRTV-seropositive samples) coming from the Central region of Virginia, in which few (N = 3) wildlife samples were available. The reason for this difference in exposure between wildlife and livestock is unclear, but it may be due to low sampling. Given the high HRTV seroprevalence in cattle, more sampling of wildlife in Central Virginia may be a future need in better understanding the exposure dynamics in that region. Further studies assessing tick-borne virus exposure in other livestock species besides cattle would also be beneficial. With the continued expansion of competent tick vector populations, understanding the potential risks to animal and human health is essential for effective prevention and control strategies.

The high tick-borne virus seroprevalence in wildlife, contrasted with the low exposure or seroprevalence in cattle, epitomizes the variation in tick access to free-living animals in an uncontrolled natural environment versus livestock confined to a controlled or limited environment. This outcome also supports the hypothesis that ticks, or perhaps primary tick species participating in viral transmission, might have more access to wildlife than livestock animals, in contrast to non-primary tick vectors. Studies elsewhere have similarly reported low livestock or domestic animal exposure to these emerging tick-borne viruses compared to wildlife. Jackson et al. [34], for example, found a BRBV seroprevalence of 4% (*n* = 24) and 15% (*n* = 13) in horses and dogs, respectively, compared to 86% (*n* = 14) in white-tailed deer and 50% (*n* = 62) in racoons. Likewise, Bosco-Lauth et al. [29] reported a seroprevalence of 42.6% (*n* = 68) against HRTV in northern raccoons, compared to 7.7% in dogs (*n* = 13). So far, wildlife seems to be more frequently exposed to POWV, BRBV, and HRTV than livestock. Moreover, cattle are frequently treated with antiparasitic medication, which could affect results; they also tend to be confined to a limited area of grassland (although variation in the wildlife–domestic interface and/or pasture tree coverage may occur), reducing their exposure to tick infestation. This study suggests that wildlife animals should be the focus of tick-borne pathogen surveillance, as they are exposed at higher rates and might potentially contribute to the geographical expansion and maintenance of tick-borne diseases in the environment.

Our study did not address a component of seasonality; however, tick-questing can occur year-round, particularly with *I. scapularis*, and the acquisition of tick-borne diseases should not be considered a purely summer risk. Climate change can extend the window of peak activity, commencing earlier in spring or continuing later into fall. Infection with tick-borne viruses should be considered a risk whenever relevant vectors are active given the propensity for the vertical transmission of tick-borne viruses [42,43,44], when vertebrates are in contact with the larval stage.

Both wildlife and livestock from the western region of Virginia, specifically the southwest and northwest, tend to be more exposed to POWV, BRBV, and HRTV than other regions of the Commonwealth. This may be due to topographical, climatic, and geological variation between the western part of Virginia and other regions, influencing the wildlife community structure and tick populations. *Ixodes scapularis* predominates on the Appalachian ridge regions along the western edge of Virginia (compared to at lower elevations of the Piedmont), thus pointing to a higher POWV persistence in that area. Wildlife movements are uncontrolled, and to some degree, exposure might have occurred elsewhere. Nevertheless, most wildlife species in the study have relatively small home-ranges; thus, it is most likely that exposure occurred in a region nearby where they were sampled. Animals in the Central and Eastern regions might also be exposed to these viruses but are not being detected due to the lower sampling there. To address this gap, serological surveillance could be continued in those regions to reveal evidence of POWV, BRBV, and HRTV circulation in Central and Eastern HPRs.

## 5. Conclusions

In summary, this study has provided valuable insight into the widespread exposure of POWV, BRBV, and HRTV in Virginia. We can now assume that wild and domestic or livestock animals across the state have been potentially exposed to all three tick-borne viruses, and this may pose a risk to public health in all HPR regions. The detection of serological evidence in a wide range of species has expanded our understanding of vertebrate exposure to tick-borne viruses and the distribution of these agents in Virginia, particularly confirming exposure to HRTV for the first time. Furthermore, the study highlights the need for vector and host studies in better understanding the ecology of these viruses, as well as understanding species’ potential to act as amplification or reservoir hosts. The recognition that three tick-borne viruses now circulate in Virginia is the first step towards developing effective strategies for limiting the disease risk from these pathogens.

## Figures and Tables

**Table 1:** Overall Seroprevalence of POWV, BRBV, and HRTV in Wildlife Samples.

Tick-Borne Virus	Number Tested	Confirmed Seropositive (%; 95% CI)	Sero-Titer
Powassan virus	811	144 (18; 15–20)	1:40–≥1:320
Bourbon virus	770	61 (8; 5.6–10)	1:40–≥1:320
Heartland virus	766	38 (5; 3.4–6.5)	1:40–≥1:320

**Table 2:** Overall Seroprevalence of POWV, BRBV, and HRTV in Cattle Serum Samples.

Tick-Borne Virus	Number Tested	Confirmed Seropositive (%; 95% CI)	Sero-Titer
Powassan virus	500	5 (1; 0.1–1.9)	1:40–≥1:320
Bourbon virus	500	6 (1.2; 0.2–2.2)	1:40–≥1:320
Heartland virus	500	40 (8; 5.6–10)	1:40–≥1:320

**Table 3:** Species of tested Wildlife showing Specific Neutralizing Antibodies against POWV, BRBV, or HRTV respectively.

Wildlife Species	Number Tested (POWV Seropositive)	POWV Seroprevalence (%; 95% CI) (Where N > 5)
White-tailed deer (*Odocoileus virginianus*)	255 (127)	49.8; 44–56
Northern raccoon (*Procyon lotor*)	111 (3)	2.7; 0–5.7
Eastern cottontail (*Sylvilagus floridanus*)	67 (1)	1.5; 0–4.4
Red fox (*Vulpes vulpes*)	40 (1)	2.5; 0–7.3
Virginia opossum (*Didelphis virginiana*)	86 (7)	8.1; 2.4–13.9
Great horned owl (*Bubo virginianus*)	4 (1)	25; 0–67 *
American toad (*Anaxyrus americanus*)	1 (1)	100 *
Eastern chipmunk (*Tamias striatus*)	1 (1)	100 *
Grey fox (*Urocyon cinereoargenteus*)	1 (1)	100 *
Eastern red bat (*Lasiurus borealis*)	2 (1)	50 *
**Wildlife Species**	**Number Tested (BRBV Seropositive)**	**BRBV Seroprevalence (%)** **(Where N > 5)**
White-tailed deer (*Odocoileus virginianus*)	249 (33)	13.3; 9–17.5
Red fox (*Vulpes vulpes*)	40 (5)	12.5; 2.3–22.7
American black bear (*Ursus americanus*)	4 (1)	25; 0–67 *
American beaver (*Castor canadensis*)	2 (2)	100 *
Striped skunk (*Mephitis mephitis*)	14 (1)	7.1; 0–20.6
Eastern cottontail (*Sylvilagus floridanus*)	68 (3)	4.4; 0–9.3
Virginia opossum (*Didelphis virginiana*)	63 (1)	1.6; 0–4.7
Groundhog (*Marmota monax*)	3 (2)	66.7 *
Northern raccoon (*Procyon lotor*)	112 (13)	11.6; 5.7–17.5
**Wildlife Species**	**Number Tested (HRTV Seropositive)**	**HRTV Seroprevalence (%)** **(Where N > 5)**
White-tailed deer (*Odocoileus virginianus*)	247 (21)	8.5; 5–12
Northern racoon (*Procyon lotor*)	112 (12)	10.7; 5–16.4
American black bear (*Ursus americanus*)	6 (2)	33.3; 0–71
American beaver (*Castor canadensis*)	2 (1)	50 *
Striped skunk (*Mephitis mephitis*)	14 (1)	7.1; 0–20.6
North American river otter (*Lontra canadensis*)	1 (1)	100 *

* = Number tested is less than five.

## Data Availability

Data are contained within the article and Supplementary Materials.

## Supplementary Materials

The following supporting information can be downloaded at: https://www.mdpi.com/article/10.3390/microorganisms12050899/s1, Table S1: List of species names of all wildlife individuals sampled during the study; Table S2: Tick-borne virus-serostatus in wildlife samples from each Health Planning Region of Virginia; Table S3: Tick-borne virus-serostatus in livestock samples from each Health Planning Region of Virginia.
