# Supplementary material for: Widespread Circulation of Tick-Borne Viruses in Virginia—Evidence of Exposure to Heartland, Bourbon, and Powassan Viruses in Wildlife and Livestock
Source: Microorganisms. 2024 Apr 30;12(5):899. doi: 10.3390/microorganisms12050899 (PMC11124039; doi:10.3390/microorganisms12050899)
Supplement: Supplementary file 1 [file microorganisms-12-00899-s001.zip › microorganisms-2925000-supplementary.pdf]

Table S1: List of species of all wildlife individuals sampled during the study. Note that, due to sample volume, not all individuals could be screened for exposure against all three tick-borne viruses.

**Common name**	**Species name**	**Available number**
American robin	*Turdus migratorius*	1
American beaver	*Castor canadensis*	2
American black bear	*Ursus americanus*	8
American crow	*Corvus brachyrhynchos*	4
American toad	*Anaxyrus americanus*	1
Bald eagle	*Haliaeetus leucocephalus*	1
Barred owl	*Strix varia*	6
Big brown bat	*Eptesicus fuscus*	2
Black vulture	*Coragyps atratus*	1
Blue jay	*Cyanocitta cristata*	2
Canadian goose	*Branta canadensis*	8
Chipmunk	*Tamias striatus*	1
Common grackle	*Quiscalus quiscula*	3
Common raven	*Corvus corax*	1
Cooper's hawk	*Accipiter cooperii*	2
Coyote	*Canis latrans*	4
Eastern box turtle	*Terrapene carolina*	5
Eastern cottontail	*Sylvilagus floridanus*	114
Eastern gray squirrel	*Sciurus carolinensis*	31
Eastern mole	*Scalopus aquaticus*	1
Eastern red bat	*Lasiurus borealis*	2
Eastern screech-owl	*Megascops asio*	1
Great blue heron	*Ardea herodias*	2
Great horned owl	*Bubo virginianus*	4
Grey fox	*Urocyon cinereoargenteus*	2
Groundhog	*Marmota monax*	17
Horned grobe	*Podiceps auritus*	1
House mouse	*Mus musculus*	5
Mallard	*Anas platyrhynchos*	1
Meadow vole	*Microtus pennsylvanicus*	7
Mourning dove	*Zenaida macroura*	1
North American river otter	*Lontra canadensis*	1
Northern red-bellied cooter	*Pseudemys rubriventris*	1
Norway rat	*Rattus norvegicus*	3
Porcupine	*Erethizon dorsatum*	2

Northern raccoon	*Procyon lotor*	117
Red fox	*Vulpes vulpes*	40
Red-shouldered hawk	*Buteo lineatus*	5
Red-tailed hawk	*Buteo jamaicensis*	5
Ring-billed gull	*Larus delawarensis*	1
Striped skunk	*Mephitis mephitis*	22
Snapping turtle	*Chelydra serpentina*	3
Turkey vulture	*Cathartes aura*	1
Virginia opossum	*Didelphis virginiana*	86
White-footed mouse	*Peromyscus leucopus*	36
White-tailed deer	*Odocoileus virginianus*	270
Wild turkey	*Meleagris gallopavo*	1
Wisconsin turtle	*Chrysemys picta*	1

Table S2: Tick-borne virus-serostatus in wildlife samples from each Health Planning Region of Virginia

**Health Region**	**Number tested**	**POWV-seropositive samples n (%; 95% CI)**
Northwestern	429	23 (5.4; 3.2-7.5)
Northern	164	6 (3.7; 0.8-6.5)
Southwestern	212	114 (54; 47-60)
Central	3	0 (0; 0-0)
Eastern	3	1 (33; 0-87)
**Health Region**	**Number tested**	**BRBV-seropositive samples n (%; 95% CI)**
Northwestern	423	47 (11; 8.1-14.1)
Northern	165	6 (3; 0.4-5.6)
Southwestern	176	8 (4.5; 1.5-7.6)
Central	3	0 (0;0-0)
Eastern	3	0 (0;0-0)
**Health Region**	**Number tested**	**HRTV-seropositive samples n (%; 95% CI)**
Northwestern	422	20 (4.7; 2.7-6.8)
Northern	164	3 (1.8; 0-3.9)
Southwestern	174	13 (7.4; 3.6-11.4)
Central	3	1(33%; 0-87)
Eastern	3	1 (33%; 0-87)

Table S3: Tick-borne virus-serostatus in livestock samples from each Health Planning Region of Virginia

**Health Region**	**Number tested**	**Number of POWV-seropositive Samples (%; 95% CI)**	**Number of BRBV-seropositive samples (%; 95% CI)**	**Number of HRTV-seropositive samples (%; 95% CI)**
Northwestern	116	1 (0.9; 0-2.5)	3 (2.6; 0-5.5)	9 (7.8; 2.9-12.6)
Northern	17	0 (0; 0-0)	0 (0; 0-0)	1 (5.9; 0-17.1)
Southwestern	173	4 (2.3; 0-4.6)	0 (0; 0-0)	4 (2.3; 0-4.6)
Central	164	0 (0; 0-0)	2 (1.2; 0-2.9)	24 (14.6; 9.2-20)
Eastern	30	0 (0; 0-0)	1 (3.3; 0-9.8)	2 (6.6; 0-15.6)
**Total**	500	5 (1; 0.1-1.9)	6 (1.2; 0.2-2.2)	40 (8; 5.6-10)
